# Lost to translation: How design factors of the mouse-tracking procedure impact the inference from action to cognition

**DOI:** 10.3758/s13414-019-01889-z

**Published:** 2019-11-05

**Authors:** Tobias Grage, Martin Schoemann, Pascal J. Kieslich, Stefan Scherbaum

**Affiliations:** 1 Department of Psychology, Technische Universität Dresden, Dresden, Germany; 2 Department of Psychology & Mannheimer Zentrum für Europäische Sozialforschung (MZES), School of Social Sciences, University of Mannheim, Mannheim, Germany

**Keywords:** Mouse tracking, Action dynamics, Boundary conditions, Experimental design, Simon task

## Abstract

From an embodiment perspective, action and cognition influence each other constantly. This interaction has been utilized in mouse-tracking studies to infer cognitive states from movements, assuming a continuous manifestation of cognitive processing into movement. However, it is mostly unknown how this manifestation is affected by the variety of possible design choices in mouse-tracking paradigms. Here we studied how three design factors impact the manifestation of cognition into movement in a Simon task with mouse tracking. We varied the response selection (i.e., with or without clicking), the ratio between hand and mouse cursor movement, and the location of the response boxes. The results show that all design factors can blur or even prevent the manifestation of cognition into movement, as reflected by a reduction in movement consistency and action dynamics, as well as by the adoption of unsuitable movement strategies. We conclude that deliberate and careful design choices in mouse-tracking experiments are crucial to ensuring a continuous manifestation of cognition in movement. We discuss the importance of developing a standard practice in the design of mouse-tracking experiments.

**Electronic supplementary material:**

The online version of this article (10.3758/s13414-019-01889-z) contains supplementary material, which is available to authorized users.

According to the embodiment stance, cognitive processing is strongly intertwined with the physical world in general, and with one’s body in particular. Consequently, cognitive processing and action are considered to constantly influence each other (see Lepora & Pezzulo, 2015; Wilson, 2002). Studies of hand motion tracking utilize this interaction by recording movements over time in order to infer the changes in cognitive states driving them (Freeman, Dale, & Farmer, 2011). Hand motion tracking has been used in many areas, for example in psycholinguistics (e.g., Bangert, Abrams, & Balota, 2012; Dale, Kehoe, & Spivey, 2007; Spivey, Grosjean, & Knoblich, 2005), value-based decision making (e.g., Dshemuchadse, Scherbaum, & Goschke, 2013; Kieslich & Hilbig, 2014), cognitive control (e.g., Erb, Moher, Sobel, & Song, 2016), distractor interference (Buetti & Kerzel, 2008, 2009; Scherbaum, Dshemuchadse, Fischer, & Goschke, 2010; Welsh, Elliott, & Weeks, 1999), and social cognition (see Stillman, Shen, & Ferguson, 2018, for a review). The most recent addition to hand motion-tracking methods is mouse tracking, the recording of hand movements via a computer mouse. This method has emerged as a seemingly simple and valuable method of processing tracing that is intuitive and easy to use for both the experimenter and the participant (see Freeman, 2018). Use of the method has hence spread widely. However, this wide and fast spread has led to many different varieties and setups of how mouse tracking is done in detail. Here we will show that such details affect how cognitive processing and action influence each other, and hence, how such details lead to different conclusions from the observed movements.

## Mouse tracking and response competition

In mouse-tracking studies, and in general in all studies of hand motion tracking, responses are not expressed through a single motoric action (e.g., a keystroke) but instead through a continuous movement (e.g., of a computer mouse’s cursor) to one of multiple response locations. This enables on-line adaptations of movement (e.g., course correction after a change of mind) during the response selection process (for a review, see Song, 2017). Movement adaptations are considered to occur because the cognitive evaluation of a choice between competing responses is constantly “leaking” into movement (Spivey, 2007; Spivey & Dale, 2006). For instance, conflicting response tendencies were studied in a Simon task with mouse tracking (Scherbaum et al., 2010; Simon, 1969) in which participants have to categorize a stimulus (i.e., a digit 1–4 or 6–9) by number magnitude (i.e., less or greater than 5) with the stimulus being presented in the vicinity of one response location. During such a binary choice task, one is usually—in early stages of processing—inclined toward the response whose location corresponds to the (task-irrelevant) stimulus position and consequently begins moving toward it. In later stages of processing, however, the (task-relevant) number magnitude is being evaluated, leading to a course correction to the other location.^1^ The resulting movement trajectory reflects the competition between the two alternatives and their dynamic changes in attraction during cognitive evaluation (Spivey & Dale, 2006; see also O’Hora, Dale, Piiroinen, & Connolly, 2013). Hence, methods like mouse tracking provide insights into cognitive processing that are not accessible by simple response time analyses. Crucially, through the continuous tracking of movement, conclusions about cognitive processes unfolding over time are made possible. This can, for instance, be used in disentangling different influences on cognitive processing by comparing their temporal signature or impact these have on mouse trajectories (e.g., Scherbaum et al., 2010; Scherbaum et al., 2016) or in dissociating different processes from one another (e.g., Dshemuchadse, Grage, & Scherbaum, 2015; Sullivan, Hutcherson, Harris, & Rangel, 2015).

## Unconsidered ramifications of design choices

Whereas the first study using a computer mouse for response selection had a more qualitative approach of analyzing the movements (Lyons, Elliott, Ricker, Weeks, & Chua, 1999), Spivey et al. (2005) were the first to combine mouse tracking with trajectory analyses, which had already been used in earlier studies of reach tracking (Howard & Tipper, 1997; Tipper, Howard, & Jackson, 1997). On the basis of these analyses, the authors inferred properties of the underlying cognitive processes, which has been done quite often ever since. However, the rise in popularity of the method (Freeman, 2018) was accompanied by a great variability in the designs of mouse-tracking experiments. In the design of a mouse-tracking study, researchers have to answer the following questions, among others: How and when should stimuli be presented? How do participants finalize their response? How much should the mouse cursor move on the screen when the mouse is moved (i.e., the mapping between hand displacement to mouse cursor displacement)? Where should responses boxes (i.e., response locations) be placed? Where should stimuli be placed? Should the available response time in a trial be limited?

The caveat about these questions is that certain design choices could blur, or even prevent, the translation from cognitive processing into mouse movements, which in turn jeopardizes valid inferences regarding cognitive processing from these movements. If, for instance, the design of an experiment would cause participants to perform most of their cognitive evaluations before movement initiation, we would expect to find mostly straight trajectories with virtually no curvature since key portions of these evaluations could not have manifested themselves in movements. The resulting trajectories would not be an accurate reflection of cognitive processing rendering valid inference difficult. In fact, Scherbaum and Kieslich (2018) were able to demonstrate in the first study investigating design factors of the mouse-tracking procedure that this can occur: The method of triggering stimulus presentation was compared between two otherwise identically designed experiments of a Simon task with mouse tracking. Most studies use—as the authors have coined it—a static starting procedure in which the stimulus is displayed automatically after a participant has clicked on a start button to initiate the trial (e.g., Dale et al., 2007). In contrast, stimulus presentation in other studies is triggered through a participant’s movement initiation (dynamic starting procedure) thereby facilitating concurrent processing and movement (e.g., Scherbaum et al., 2010). These two design options led to different movement strategies in the study: Whereas the participants in the dynamic starting procedure were forced to initiate their movement in order to trigger stimulus presentation, the participants in the static starting procedure, who were able to freely decide when to initiate their movement, often delayed movement initiation considerably, which yielded reduced consistency of movements as well as weaker effects in continuous measures (i.e., measures stretching across multiple points in time) with the static starting procedure.

In this article, we go a step further in investigating the interplay of three design factors of the mouse-tracking procedure. For this, we used the same Simon task and the static starting procedure, in order to directly expand the previous work by Scherbaum and Kieslich (2018). We studied three design factors that varied between different mouse-tracking studies—namely, response selection, hand/cursor movement ratio, and response box position. First, the method of response selection concerns the question of how participants finalize their response. In previous research, participants selected their response either by moving the mouse cursor into a response box (called the *hover condition* in the following sections, as the trial was terminated directly after moving the cursor into a response box; e.g., Duran, Nicholson, & Dale, 2017; Huette & McMurray, 2010; see Table 1) or by clicking inside a response box (click condition; e.g., Freeman, 2014; Spivey et al., 2005). The click condition has been used by a majority of studies (Schoemann, O’Hora, Dale, & Scherbaum, 2019). When employing the click response selection method, the response is not performed by mouse movements alone. The required click adds another component to the process that is not part of the movement trajectory. This component, however, could be quite important, since it allows for second guessing one’s choice: One can linger after reaching the response box and reevaluate the choice, which can even lead to a change of mind (see Barca & Pezzulo, 2015). Conversely, in the hover response selection, the decision is finalized upon reaching the response box. Thus, one is fully committed to a response when nearing a response location in the hover response selection, whereas full commitment is unnecessary in the click response selection, which we expect would facilitate a liberal way of moving in this response selection.

**Table 1:** Exemplary mouse-tracking studies utilizing different approaches with the design factors manipulated in this study

Study	Response Selection	Hand/Cursor Movement Ratio	Response Box Position
Dale et al. (2007)	Click	Not reported	Corner
Duran et al. (2017)	Hover	Not reported	Medial
Spivey et al. (2005)	Click	Not reported	Medial
Freeman (2014)	Click	0.00125 in./pixel	Corner
Huette & McMurray (2010)	Hover	Not reported	Corner

The hand/cursor movement ratio from Freeman (2014) is measured as hand displacement in inches to cursor displacement in pixels.

Second, the hand/cursor movement ratio concerns the impact of the translation of physical computer mouse movements into cursor movements. This ratio is low when a small hand movement (e.g., 1 cm) is translated into a big cursor movement (e.g., 10 cm) such that the cursor can be moved across the screen with relatively small hand movements. The higher the ratio the more hand movement is required to move the cursor across the screen (e.g., 1 cm mouse movement to 5 cm cursor movement). M. H. Fischer and Hartmann (2014) pointed out that hand/cursor movement ratios are rarely being reported and stressed the importance of doing so. They proposed to use a rather high hand/cursor movement ratio in order to achieve a close, linear connection between hand movement and mouse movement. Indeed, a low hand/cursor movement ratio might impede capturing cognitive processing accurately since small hand movements would lead to disproportionately greater mouse movements. Because of this, participants could over-adapt and move in a too-tentative manner, which in turn would lead to a reduction in effects of action dynamics. Another possible consequence of low hand/cursor movement ratio, however, is that participants are able to adapt to the ratio by moving the hand in smaller amounts. In this case, the hand/cursor movement ratios would have no effect on mouse trajectories.

**Fig. 1:**
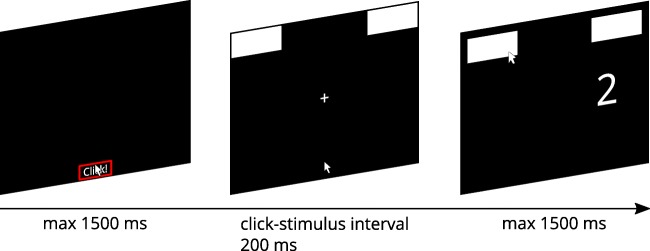
Setup of the experiment. At the beginning of each trial, the participant had to click inside the start box at the bottom center of the screen. After that, the response boxes were presented at the top of the screen. The boxes were either positioned in the corners of the screen (second panel) or shifted toward the screen center (third panel). Stimulus onset followed a constant click–stimulus interval of 200 ms. After stimulus onset, the participant had to respond to a number magnitude by moving inside the appropriate response box (hover condition) or clicking inside the appropriate response box (click condition).

Third, the response box location concerns the impact of the eccentricity of response boxes on mouse trajectories. In a majority of studies (Schoemann, O’Hora, et al., 2019), response boxes are located directly in the top corners of the screen (e.g., Dale et al., 2007; Huette & McMurray, 2010). In other studies (e.g., Faulkenberry & Rey, 2014; Spivey et al., 2005) however, boxes are detached from the screen’s corner and placed more toward the center thereby creating a small gap between the screen’s border and the response box (see Fig. 1)*.* We call these placements corner and medial response box positions, respectively. Corner response boxes can be hit easily as long as participants move the mouse upward as well as either left or right, which allows for a movement requiring only a small amount of planning and focus. In contrast, medial response box locations require participants to aim more precisely for the response boxes and might hence increase the necessary amount of planning and focus. One could furthermore expect an interaction of this design factor with the response selection design factor: Differences between both response box positions should especially affect participants in the click response selection group as they additionally have to pay attention not to overshoot the response box before clicking.

## Hypotheses

Our main hypothesis concerned the design factor response selection: We hypothesized (1a) that the click response selection (due to its weakened relationship between distance to a response and commitment to it) causes weaker continuous effects—that is, a weaker contrast between incongruent and congruent trials of mouse cursor trajectories, in comparison to the hover response selection. We further expected (1b) that underlying these weaker effects is a reduced movement consistency in the click response selection as expressed by higher variance in mouse movements and a discontinuous velocity profile.

Our secondary hypotheses concerned the remaining two design factors: For the design factor response box position, we expected (2a) weaker continuous effects when boxes are detached from the screen’s corners (i.e., medial response box positions) due to the higher demand in movement planning. This should especially be the case in the click response selection. Furthermore, we expected (2b) that the increased demand in movement planning results in reduced velocities of mouse movements. Finally, for the design factor hand/cursor movement ratio (2c), we currently can only speculate on the basis of an experimenter’s experience, since this factor is rarely reported, and only anecdotal evidence exists so far. Hence, we opted for an exploratory approach based in the following reasoning: If participants are unable to adapt to a low hand/cursor movement ratio, we would expect exaggerated movements, which in turn could lead to larger continuous effects. If participants over-adapt to a low ratio, we would expect the opposite—that is, weaker continuous effects.

## Method

### Participants

The study was performed in accordance with the Declaration of Helsinki and the ethical principles of the German Psychological Society. Approval by the local ethics committee was not required, since the study did not involve any risk or discomfort for the participants. All participants were informed about the purpose and the procedure of the study and gave written informed consent prior to the experiment. All data were analyzed anonymously.

The study by Scherbaum et al. (2010) had a sample size of 20 participants. Since we were using an additional between-subjects manipulation (i.e., response selection), we recruited 40 volunteers, who took part in the experiment at Technische Universität Dresden and were randomly assigned to either the click condition or the hover condition. One participant withdrew consent after the experiment was finished, and another one was excluded from the analyses due to an exceedingly high error rate of 43.7% (5.4 standard deviations above the sample mean). Thus, the final sample consisted of 38 participants (18–38 years of age, mean = 24.6, *SD* = 4.7). In all, 60.5% of the participants were female, 39.5% male. A total of 35 participants reported being exclusively right-handed, two participants were mostly right-handed, and one participant was mostly left-handed. Every participant performed the task with the right hand. All participants had normal or corrected to normal vision. They received €5 payment.

### Apparatus and stimuli

The experiment was presented using MATLAB (The Mathworks, Natick, MA, USA) and Psychtoolbox (Brainard, 1997; Kleiner, Brainard, & Pelli, 2007; Pelli, 1997) on a personal computer with Windows XP (Service Pack 2) and displayed on a 19-in. CRT monitor (1,280 × 1,024 pixel resolution, 85-Hz refresh rate, viewing distance 60 cm). We used a wired laser computer mouse (Logitech RX1500; sampling rate: 92 Hz; resolution: 1,000 dpi) to record mouse movement trajectories.

The stimuli used were the digits 1–4 and 6–9, which were displayed 400 pixels (11.8 cm) to the right or left of the screen’s center. They had a width of 140 pixels (4.1 cm) and a height of 190 pixels (5.6 cm). The widths of the response boxes and the start box were 224 pixels (6.6 cm) and 140 pixels (4.1 cm), respectively. The minimal distance between start box and response boxes (placed in the screen’s corners) was 1,158 pixels (34.1 cm).

### Procedure

At the beginning of each trial, participants had to click inside the start box at the bottom center of the screen within a deadline of 1,500 ms, causing the response boxes and a fixation cross to appear (Fig. 1). After a set click–stimulus interval of 200 ms, the stimulus appeared. The stimulus appeared on the left-hand or right-hand side of the screen. Participants were instructed to respond to number magnitude of a digit by moving the cursor into the upper left response box for digits less than five and into the upper right response box for digits greater than five, regardless of stimulus position. They were also instructed to move upward without stopping or moving backward, thereby encouraging the participants to create a mouse trajectory that captures the entire decisional process. Additionally, participants who were in the click condition had to click inside the appropriate response box to indicate their response and were instructed accordingly. All participants had to respond within an interval of 1,500 ms. If any deadline was missed, the trial was aborted, the participant received feedback about this, and the next trial began.

Participants received onscreen instructions and a demonstration of the mouse-tracking procedure by the experimenter. They practiced the task in 40 trials (ten trials with response feedback and without deadlines, ten trials with response feedback and deadlines, and 20 trials under the conditions of the actual experiment—i.e., without response feedback and with deadlines).

### Design

A mixed-factor 2 × 2 × 2 design was used. Response selection (hover, click) was the between-subjects factor; hand/cursor movement ratio (low, high) and response box position (corner, medial) were within-subjects factors.

To switch between hand/cursor movement ratios, we used the Mouse Acceleration Toggler (Version 1.0.3.1; Holmes, 2010). We set speed values of 6 and 9 in this software for the high and low hand/cursor movement ratios, respectively (this corresponds to 30% and 45% of the maximum Windows Pointer Speed in the system settings, respectively). The resulting hand/cursor movement ratios (measured manually) were 0.159 cm of hand movement per centimeter cursor movement for the high ratio, and 0.09 cm of hand movement per centimeter cursor movement for the low ratio (an increase of 175.8%).^2^ Pixel measurements were obtained with the Pixelruler software (Version 9.5.0.0; Rosenbaum, 2017). Nonlinear cursor acceleration (“Enhance pointer precision” in the system settings) was turned off.

The response box positions were either set to the corners of the screen (corner) or indented toward the screen’s center (medial). The amount of indention emulated the response box positions used by Spivey et al. (2005). Specifically, the response boxes were shifted by 88 pixels (2.6 cm) horizontally and by 52 pixels (1.5 cm) vertically (see Fig. 1).

The experiment was divided into four blocks with 256 trials each. Within each block, we varied systematically for each trial the target direction (as indicated by number magnitude; left, right), target position (left, right) of the current trials with respect to the target direction and target position of the previous trial. We balanced the sequence of trials via pseudo randomization resulting in a transition matrix of Trial_*N*_ (4) × Trial_*N*–1_ (4) × Trial Repetition (16) for each block. Through this, the congruency of a target’s direction and position in the previous and current trials, as well as response repetition and stimulus transitions, was systematically varied, and hence controlled for.

Response box position and hand/cursor movement ratio were varied blockwise. The order of changes was counterbalanced across participants. Starting with the second block, the participants were informed at the beginning of each block about what change regarding these factors were to occur (e.g., “The position of the response boxes has changed.”) and were asked to perform ten practice trials to get accustomed to it.

### Data preprocessing and statistical analyses

#### Data exclusion

We excluded erroneous trials (2.18%), trials following errors, and trials with response times greater than 2.5 standard deviations of a participant’s mean (0.72%) from analysis. All mouse trajectories were horizontally realigned to have a common starting position at the horizontal center of the screen. Mouse trajectories to the right response box were mirrored horizontally, so that for each trial the left response box represented the correct answer. Response times were calculated as the duration between stimulus onset and reaching or clicking into a response box (for hover and click conditions, respectively).

#### Calculation of temporal measures

The different response conditions (click vs. hover) asked for an additional data processing step for the click condition: In the hover condition, crossing the border of a response box ended the trial immediately. In contrast, in the click condition, participants could traverse both response boxes and continue moving inside them without the trial being terminated. To make measures between the two conditions comparable, all trajectories of the click group were cut at the last crossing of a response box’s border for each trial. On average, the participants in the click condition spent 128.2 ms (*SD* = 27.2 ms) within the response box after crossing its boundary. This was equivalent to 17.2% (*SD* = 3.3%) of the response times spent within the response boxes. Response times were recalculated for the click condition accordingly. The mouse trajectories of both groups were normalized afterward to 100 equally sized time slices (Scherbaum et al., 2010; Spivey et al., 2005).

#### Statistical analyses

If not otherwise specified, the significance level used was *α* = .05.

Data preprocessing and aggregation were performed in Matlab (The Mathworks, Natick, MA, USA). Statistical analyses were performed in Matlab and JASP (JASP Team, 2019).

## Results

We will first present the results for the design factor response selection: (i) the continuous effects for both the hover and the click group, and (ii) the results for the quality of mouse movements—namely, movement consistency, movement density, and discreteness of movements. Second, we will present the results for movement initiation time analyses. Subsequently, the results for the design factors hand/cursor movement ratio and response box position are presented.

As a manipulation check, we tested the occurrence of the Simon effect in an analysis of variance (ANOVA) of response times that yielded significance across all design factors. These and other additional analyses are being reported in the supplementary material.

### Response selection

#### Continuous effects

Hypothesis 1a stated that the click response selection would lead to weaker continuous effects than the hover response selection. For this, we analyzed mouse movements on the horizontal axis (i.e., *x*-movements) and cursor velocity (i.e., Euclidean distance between each consecutive pair of two samples) as a function of time: To determine differences in the Simon effect in *x*-movements, we calculated and tested contrasts of mouse trajectories (see Dshemuchadse et al., 2013): For both conditions, we subtracted averaged *x*-movements of incongruent from congruent trials. Then, we tested the difference between design factor levels (i.e., click and hover) with *t* tests for each time step. To correct for multiple comparisons of temporally dependent measures, *α* was set to .01 and only sequences of at least eight consecutive and significant *t* tests were included (see Dale et al., 2007).

**Fig. 2:**
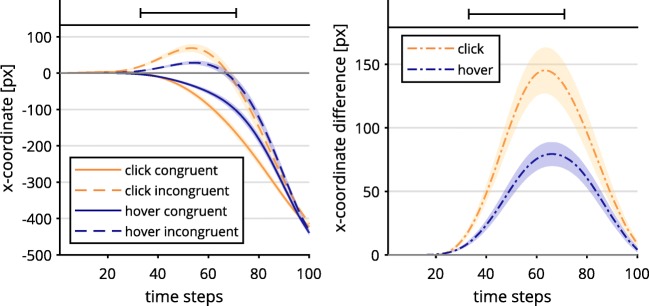
Mouse trajectory data along the *x*-axis per time-normalized step of incongruent and congruent trials, depending on response selection (left) and incongruent–congruent contrast (right). Lines above the graphs mark time steps with significantly different contrasts (*α* = .01; see the main text for statistical details). The trajectory data were first averaged within and then across participants. Shaded confidence bands depict standard errors.

Contrary to Hypothesis 1a, the click condition exhibited a significantly stronger Simon effect in *x*-movement than did the hover condition, occurring at Time Steps 33–71, all *t*s(36) ≥ 2.73, all *p*s < .01. This was driven by a stronger erroneous movement tendency in incongruent trials and a more direct approach in congruent trials (see Fig. 2)*.*

Regarding the velocity of mouse movements, we found differences between the click group and the hover group regardless of congruency. We calculated independent samples *t* tests for each time step and corrected for multiple comparisons as described before.

**Fig. 3:**
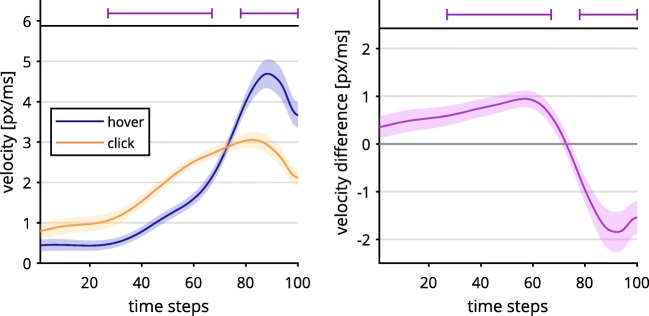
Velocity data per time-normalized step depending on response selection (left), and contrasted trajectory data (right). Lines above the graphs mark time steps with significantly different contrasts (*α* = .01). The trajectory data were first averaged within and then across participants. Shaded confidence bands depict standard errors.

For the hover condition, we found an inconsistent and rather ballistic velocity profile of starting out slow and accelerating to high speeds in the last two thirds of trials (see Fig. 3). The click condition exhibited a more stable speed profile, with less acceleration involved. As such, hover was significantly slower for Time Steps 27–67, and faster for time steps 78–100, all |*t*s(36)| ≥ 2.76, all *p*s < .01. This contradicted part of Hypothesis 1b, which stated that the click group should exhibit a discontinuous velocity profile.

Taken together, in the click group we found stronger action dynamics for the Simon effect in *x*-movements than in the hover group, which contradicted Hypothesis 1a, predicting weaker continuous effects for the former group. The contrasts of velocity profiles revealed rather unstable, ballistic velocities in the hover as compared to the click condition, also controverting Hypothesis 1b.

#### Close inspection via heatmaps

**Fig. 4:**
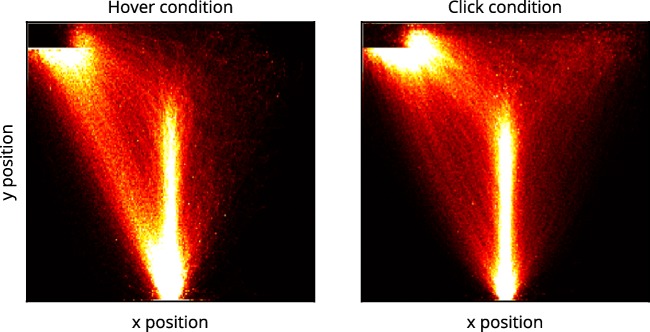
Heatmaps of pooled mouse movements on the *xy*-plane for the hover condition (left) and the click condition (right). Mouse movements begin in the start box at the bottom center and terminate in the response box in the top left corner. Brighter colors depict a higher frequency of mouse movement in the corresponding area. Mouse movements for trials with medial boxes were scaled up so that the medial response box position was aligned with the corner response box position.

To better understand these unexpected results, we took a closer look at the way participants moved. For this analysis, we calculated heatmaps of pooled mouse movements on the *xy*-plane that showed the frequency with which mouse movements passed through binned areas of the screen. The patterns of mouse movement density were substantially different between the hover condition and the click condition (Fig. 4): First, the click condition exhibited an overall higher spread in movement density and higher movement density in the vicinity of the incorrect response box than did the hover condition. Second, the hover condition appeared to branch out at the starting area into a direct route and a route starting with a straight upward movement.

We expected the movement pattern for the click condition in Hypothesis 1b. However, this should have led to a reduction in continuous effects, as stated in Hypothesis 1a. This pattern could indicate that participants moved directly to the incorrect response box and only then corrected their decisions—a phenomenon that will be referred to as *discrete changes of mind* in the following discussion (abbreviated dCOMs; see also Wulff, Haslbeck, Kieslich, Henninger, & Schulte-Mecklenbeck, 2019). The pattern found in the hover condition could indicate that participants followed different movement strategies. We investigated both observations thoroughly, as we report below.

#### Discrete changes of mind

**Fig. 5:**
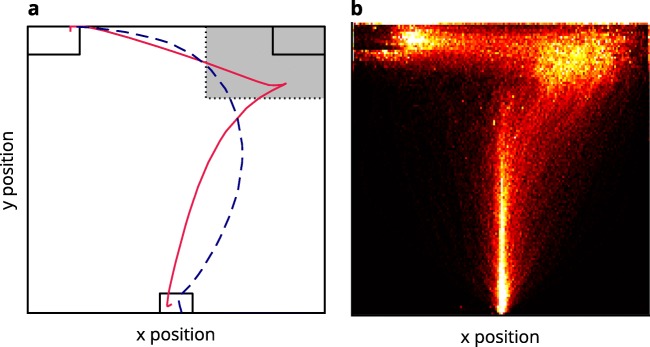
Classification of discrete changes of mind (dCOMs). (A) The experiment’s screen, with the shaded gray area depicting the area of interest for classification of trials that include a dCOM. Two exemplary, empirical movement trajectories are displayed. The solid trajectory was classified as a dCOM, whereas the dashed trajectory was not, since the rate of change in cursor angle was too small. (B) Heatmap of pooled mouse movements for the classified trials in the click group.

A dCOM results in a trajectory shape that is characterized by a direct movement toward the unchosen option, followed by a horizontal movement to the chosen response box (see Wulff et al., 2019). These trajectories are problematic for using mouse movements to trace cognitive processes, as they indicate that the movement does not reflect continuous competition manifesting itself in movement, but instead indicates the sequential execution of two processes that lead to two relatively discrete responses. To determine the proportion of trials featuring dCOMs in our data, we classified a trial as such if it comprised a strong change in mouse cursor angle toward the correct response occurring in the top right quadrant of the screen (i.e., the area of the incorrect response box). For this, we estimated each trajectory with a cubic smoothing spline, which was then differentiated to determine rate of change. Only parts of the sample situated in the top 25% of the screen and the rightmost 40% were inspected. If the rate of cursor angle change in this area was smaller than – 0.1, the trajectory was classified as including a dCOM^3^ (Fig. 5).

**Fig. 6:**
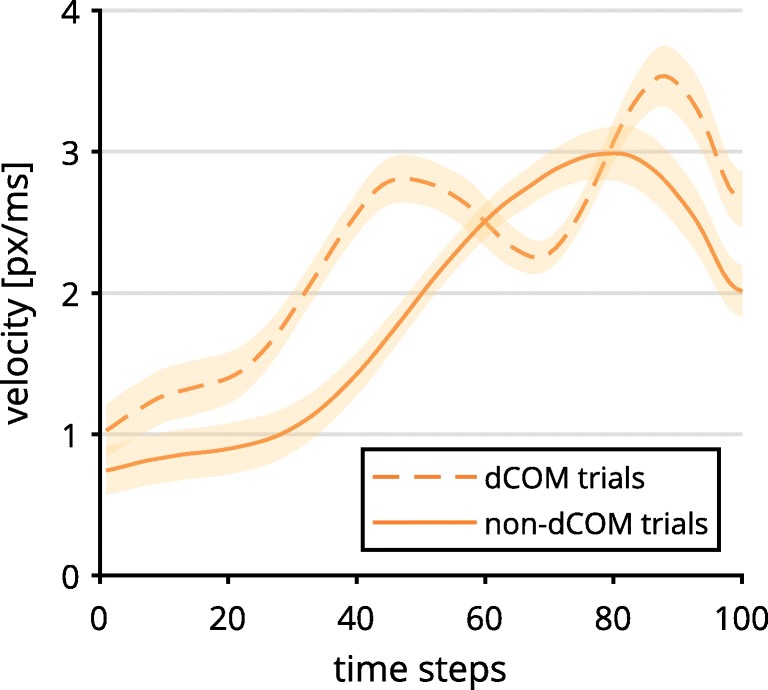
Velocity profile of discrete change of mind (dCOM) trials and non-dCOM trials in the click condition. The trajectory data were first averaged within and then across participants. Shaded confidence bands depict standard errors.

**Fig. 7:**
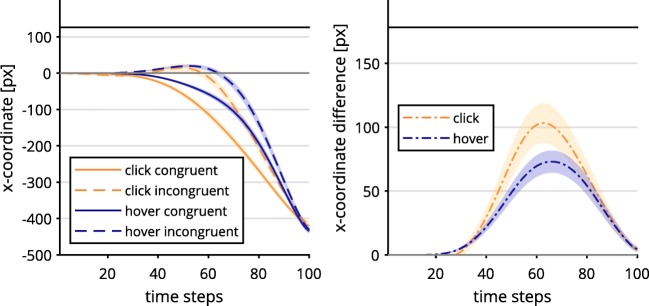
Mouse trajectory data along the *x*-axis per time-normalized step for incongruent and congruent trials, depending on response selection (left) and incongruent–congruent contrast (right). The trajectory data were first averaged within and then across participants. Shaded confidence bands depict standard errors. All trials with discrete changes of mind have been eliminated from the analysis.

The frequency of dCOM trials was significantly higher across participants in the click group (*M* = 15.0%, *SD* = 13.7%) than in the hover group (*M* = 3.4%, *SD* = 3.5%), *t*(20.35) = 3.55, *p* = .002, *d* = 1.15, 95% CI [0.42, 1.87]. The average trajectories of dCOM trials in the click group exhibited a different velocity profile from non-dCOM trajectories (Fig. 6): Trials with dCOMs started out faster than non-dCOM trials and included two distinct velocity peaks, between which the direction of movement was changed. Since dCOM trials lead to high deviations of mouse movements, we reasoned that differences between the hover and click conditions might simply be a result of the higher number of dCOM trials in the click condition. We repeated the analysis of the Simon effect in continuous *x*-movements (see Fig. 2), but this time excluding all dCOM trials from the analysis (Fig. 7). The exclusion of dCOM trials indeed changed the observed action dynamics in the click condition, so that the difference between the incongruent–congruent contrasts in the click and hover conditions was no longer significant. These findings could explain the unexpectedly high continuous effects in the click condition, which contradicted Hypothesis 1a.

#### Movement strategies

**Fig. 8:**
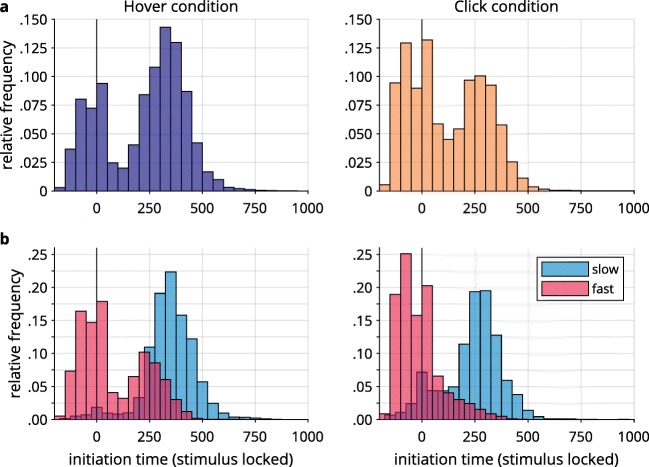
(A) Relative frequency distribution of movement initiation times, in milliseconds, relative to the time of stimulus onset (i.e., relative to 200 ms after the start of a trial), separated for the hover (left) and click (right) conditions. (B) Relative frequency distributions for response selection groups after a median split by movement initiation time, splitting the groups into fast- and slow-initiating subgroups.

Movements in the hover condition split into a direct route and a curved route (Fig. 4 left). We suspected that the branching off into a direct path and a more indirect and curved path was the result of different strategies employed by the participants: Since participants were able to initiate their movement self-paced, they could have delayed movement until they had determined the correct response. This would be problematic for using mouse movements to trace cognitive processing, as most of cognitive processing would be already completed before the mouse was moved by a participant, leading to relatively discrete responses. Thus, we assumed that long movement initiation times were associated with quick and direct mouse movements, reflecting high levels of commitment toward a response. To investigate this, we calculated movement initiation times by determining the point in time at which participants moved the mouse cursor in two consecutive samples by at least four pixels each. This was the method employed by Scherbaum et al. (2010) to trigger stimulus onset in their dynamic start condition.^4^ Figure 8 shows histograms of the pooled movement initiation times, relative to stimulus onset, that occurred statically 200 ms after the beginning of a trial. Initiation times comprised a broad range across participants in both the hover (*M* = 220.9 ms, *SD* = 190.9 ms) and the click (*M* = 119.1 ms, *SD* = 176.1 ms) conditions. Premature movement initiation (i.e., initiation times < 0, before stimulus onset) was more pronounced in the click condition (31.9% of trials) than in the hover condition (19.2%). In several of these trials, participants moved significant distances toward the top of the screen before stimulus onset occurred (see the supplementary material). Incidentally, dCOM trials had faster initiation times than did non-dCOM trials in both the hover (non-dCOM: *M* = 220.5 ms, *SD* = 165.9 ms; dCOM: *M* = 150.1 ms, *SD* = 133.8 ms) and the click (non-dCOM: *M* = 125.5 ms, *SD* = 148.0 ms, dCOM: *M* = 70.1 ms, *SD* = 117.5 ms) conditions, both *t*s(18) ≥ 6.06, both *p*s < .001, both *d*_*z*_ ≥ 1.39. In contrast, in the hover condition, trials had a greater tendency to start late (e.g., ≥ 250 ms after stimulus onset: 54.5% of trials) than in the click condition (29.5%).

**Fig. 9:**
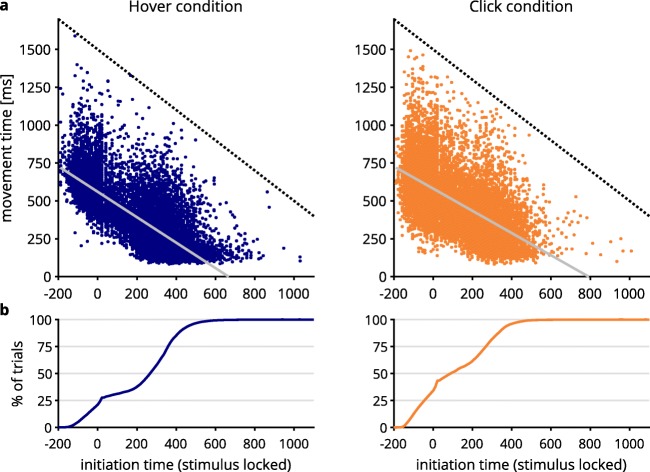
(A) Scatterplots of initiation time and movement time, separated for hover (left) and click (right) response selection (pooled data). (B) Cumulative percentages of trials are shown as a function of initiation time. The dotted black lines in row A depict the upper limit for combinations of initiation and movement time of 1,700 ms (1,500-ms trial deadline plus 200-ms stimulus onset). Because of this, initiation time and movement time were inevitably interlinked at the higher end of response times. However, the relationship between movement and initiation time was rarely driven by this upper limit. The solid gray lines in row A depict least-squares fits.

The assumption that long initiation times were associated with quick movements was confirmed by significant negative correlations across participants between mean initiation time and mean movement time (i.e., response time minus initiation time) in both the hover group, *r* = – .96, 95% CI [– .90, – .98], *p* < .001, and the click group, *r* = – .84, 95% CI [– .62, – .94], *p* < .001 (see Fig. 9 for the impact of initiation time on trial level).^5^ Confidence intervals indicate that the correlation was stronger for the hover group.

The considerable variance in initiation times, together with the negative correlation of initiation times and movement times, indicates that movement initiation strategies varied interindividually: One extreme combined short initiation times (and hence, a short time for cognitive processing before movement) with long movement times (and hence, ample opportunity for cognitive processing to leak into the movement). The other extreme combined long initiation times (and hence, ample opportunity for cognitive processing before movement) with short movement times (and hence, much less opportunity for cognitive processing to leak into movements). For this reason, we divided both response selection groups separately into subgroups by performing a median split with slow and fast movement initiation times (see Vogel, Scherbaum, & Janczyk, 2018). The median initiation times were 282.2 ms for hover and 81.8 ms for click (see Table 2 for the means and standard deviations of initiation times in the resulting subgroups; see also Fig. 8B).

**Table 2:** Means and standard deviations of movement initiation time, after a median split for separate slow- and fast-initiation subgroups in both the hover and click conditions

Subgroup	Hover		Click	
	*M*	*SD*	*M*	*SD*
Fast initiation	79.7	153.7	– 13.5	104.0
Slow initiation	345.7	120.8	239.0	137.8

All means and standard deviations are in milliseconds.

**Fig. 10:**
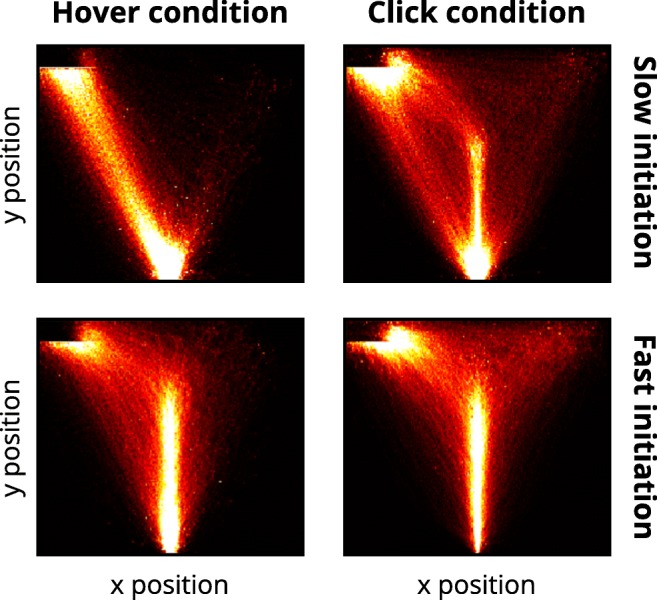
Heatmaps of pooled mouse movements on the *xy*-plane for the hover condition (left) and the click condition (right). Separate plots are shown for movement initiation time subgroups: slow initiation times (top) and fast initiation times (bottom). Mouse movements begin in the start box at the bottom center and terminate in the response box in the top left corner. Brighter colors depict higher frequencies of mouse movement in the corresponding area. Mouse movements for trials with medial boxes were scaled up so that the medial response box position was aligned with the corner response box position, for reasons of comparability.

Heatmaps of mouse movement density on the *xy*-plane were generated for each subgroup and both response selection methods (see Fig. 10). For the hover condition, participants with slow initiation times performed straight movement trajectories almost exclusively, whereas participants with fast initiation times performed curved movements on average. For the click condition, the differences between slow and fast movement deviations were not as pronounced. Nevertheless, participants with slow initiation times exhibited a distinct density profile, which comprised a direct route, a curved route, and some movements deviating strongly toward the wrong response box. The spreading of movements occurred directly at the start box, unlike the top-heavier spread of participants with fast initiation times. The latter group did not exhibit a direct route, but almost exclusively a curved route with a higher density of movements around the wrong response box. Overall, both slow-initiating groups demonstrated an earlier commitment toward a response alternative (which was more pronounced in the hover condition), whereas both fast-initiating groups demonstrated more spread in movement densities (which was more pronounced in the click condition).

### Response box position and hand/cursor movement ratio

We hypothesized that the medial response box position would lead to weaker continuous effects, especially in the click condition (Hypothesis 2a), and reduced velocities of mouse movements (Hypothesis 2b). For the design factor hand/cursor movement ratio, continuous effects could be either weaker or stronger, depending on whether participants were unable to adapt to a low hand/cursor movement ratio or over-adapted to it (Hypothesis 2c). For this, we again analyzed *x*-movements and cursor velocity via contrasts of the design factor levels, as described above. Inspired by the exploratory findings for the design factor response selection, we additionally investigated the effects of response box position and hand/cursor movement ratio on discrete changes of mind and on movement strategies.

#### Tests of initial hypotheses

Contrary to Hypothesis 2a, we found no significant differences between response box positions in *x*-movements in either the click or the hover group (see the supplementary material for a plot).

For the velocity of mouse movements, we found differences between design factor levels regardless of congruency. To test these differences, we subtracted the averaged mouse measures for medial from those for corner response boxes and tested whether the resulting contrasts were different from zero for each time step (see Table 3). We corrected for multiple comparisons as described before.

**Table 3:** Significant segments of contrasts in mouse cursor velocity between the secondary design factors

Contrast	Response Selection	Segments	*t*
Corner–medial boxes	Hover	72–100	*t*s(18) ≥ 2.89
	Click	6–28,	*t*s(18) ≤ – 2.88
		70–100	*t*s(18) ≥ 3.33
Low–high hand/cursor movement ratio	Hover	79–100	*t*s(18) ≥ 2.99
	Click	72–100	*t*s(18) ≥ 3.71

All *p*s < .01.

**Fig. 11:**
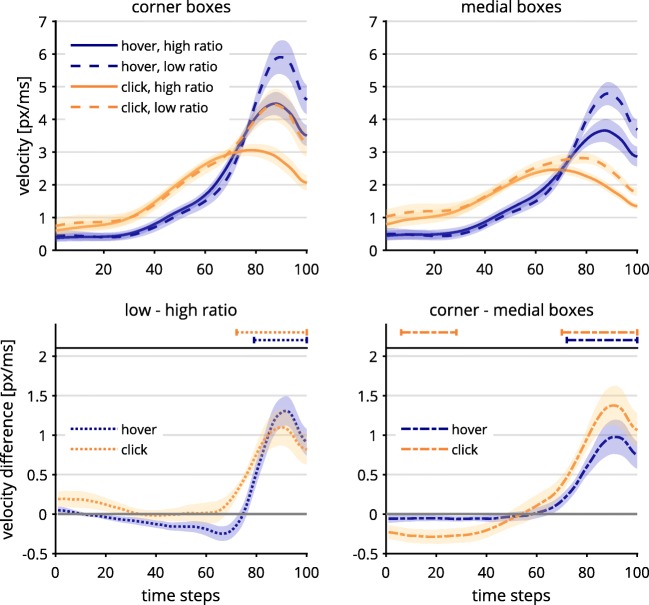
Velocity data per time-normalized step, depending on response selection and hand/cursor movement ratio and separated by response box position, either corner (top left panel) or medial (top right panel), as well as contrasted trajectory data (bottom panels). Lines above the graphs mark time steps with significantly different contrasts (*α* = .01). Trajectory data were averaged first within and then across participants. Shaded confidence bands depict standard errors.

We found higher velocities for corner response box positions in the last quarter of time steps, in comparison to medial response box positions (Fig. 11). This was where velocities were highest, which was likely due to the participants being committed to a response at this point, so they just had to follow through. The fact that velocities were lower for medial response box positions could indicate that participants indeed required more precision in order to hit these response boxes. This resulted in lower cursor velocities, corroborating Hypothesis 2b. In contrast, the initial velocities for medial response box positions (Time Steps 6–28) were significantly elevated in the click response selection. However, this difference was rather small.

For the analyses of the design factor hand/cursor movement ratio (Hypothesis 2c), we calculated and tested the contrast between low and high movement ratios in mouse cursor velocity, as described before.^6^ Low hand/cursor movement ratios led to higher velocities than did high ratios in the last quarter of time steps (see Table 3 and Fig. 11). This indicated that participants moved the mouse more slowly to compensate for the low ratio until they could commit to a response.

#### Exploratory analysis of discrete changes of mind and movement strategies

**Fig. 12:**
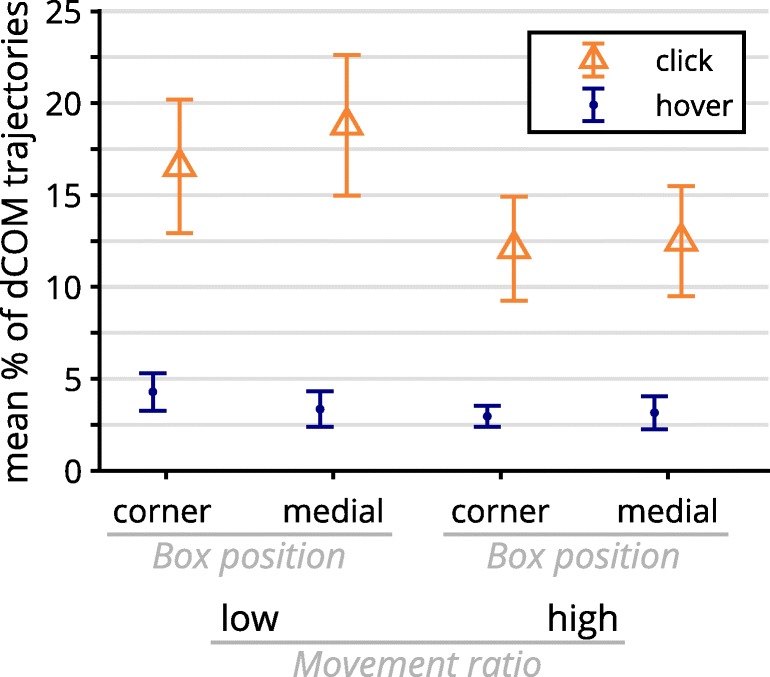
Mean frequencies of discrete changes of mind, separated for response selection, response box position, and hand/cursor movement ratio. Error bars indicate standard errors.

The effects of discrete changes of mind as well as movement strategies shed light on the unexpected dynamic effect results found in the analyses of the primary design factor response selection. To acquire a complete picture of how all design factors together influenced dCOMs, we performed an ANOVA with the factors response selection, hand/cursor movement ratio, and response box position, with the frequency of dCOM trials as the dependent variable. This analysis revealed significant main effects of response selection and hand/cursor movement ratio, as well as a significant interaction between these two factors (see Fig. 12). Regarding the response selection main effect, *F*(1, 36) = 12.58, *p* = .001, *η*_p_^2^ = .26, dCOMs were more frequent in the click group (*M* = 15.0%, *SD* = 13.7%) than in the hover group (*M* = 3.4%, *SD* = 3.5%). The significant hand/cursor movement ratio main effect, *F*(1, 36) = 12.35, *p* = .001, *η*_p_^2^ = .26, was driven by the interaction with response selection, *F*(1, 36) = 6.97, *p* = .012, *η*_p_^2^ = .16: In the click group, a low hand/cursor movement ratio led to more dCOMs (*M* = 17.7%, *SD* = 15.8%) than did a high hand/cursor movement ratio (*M* = 12.3%, *SD* = 12.4%), *t*(18) = 3.20, *p* = .005, *d*_z_ = 0.73. This effect was not significant in the hover group, *t*(18) = 1.64, *p* = .118. There were no other significant effects (all *p*s ≥ .15).

To investigate the influence of the design factors on movement strategies, we focused again on the correlation between initiation time and movement time, which had shown a negative relationship in our study of the design factor response selection. We determined this correlation again for each level of response box position and hand/cursor movement ratio (see Table 4). As indicated by the confidence intervals, neither the response box position nor the hand/cursor movement ratio led to a change in movement strategies, as indicated by their comparable correlations.

**Table 4:** Correlations of movement initiation time and movement time for each design factor level on the subject level

	Hover			Click		
Design Factor Level	*r*	95% CI	*p*	*r*	95% CI	*p*
Corner response boxes	– .95	[– .88, – .98]	< .001	– .85	[– .64, – .94]	< .001
Medial response boxes	– .96	[– .90, – .98]	< .001	– .83	[– .61, – .93]	< .001
High hand/ cursor movement ratio	– .94	[– .86, – .98]	< .001	– .80	[– .54, – .92]	< .001
Low hand/ cursor movement ratio	– .96	[– .91, – .99]	< .001	– .86	[– .66, – .94]	< .001

Correlations were calculated by correlating participants’ initiation time means with their movement time means.

Overall, regarding Hypothesis 2a, we did not find the predicted differences in action dynamics for response box locations. Regarding Hypothesis 2b, we found the expected reduction in the velocity of mouse movements for medially placed response boxes at the end of trials. Regarding Hypothesis 2c, we found evidence for both successful and unsuccessful adaptation to low hand/cursor movement ratios: Successful adaptation was indicated by mouse velocities, for which a low hand/cursor movement ratio resulted in high velocities at the ends of trials. Unsuccessful adaptation was indicated by the increased frequency of dCOM trials in the low hand/cursor movement ratio condition compared to the high hand/cursor movement ratio condition.

## Discussion

The present study investigated how three design factors of the mouse-tracking procedure influence the translation from cognitive processing into mouse movement. The studied design factors were the method of response selection (hover vs. click), hand/cursor movement ratio (low vs. high) and the position of response boxes (placed in corners vs. placed medially).

We hypothesized that these factors would have an impact on the strength of continuous effects, movement consistency, and, in turn, on different measures of effects in a cognitive paradigm, which is the Simon task (Scherbaum et al., 2010; Simon, 1969). Additionally, we performed exploratory analyses of two phenomena that might affect the validity of the assumption that cognitive processing leaks into mouse movements at all, namely discrete changes of mind and movement strategies.

### Response selection

The manipulation of the response selection led to the greatest effects among the design factors studied. In the so-called click condition, participants had to indicate their response by clicking into a response box, whereas in the so-called hover condition, they only had to move the mouse into a response box.

#### Variance in movements and discrete changes of mind

In the click condition, we found greater variance in heatmaps of movement density than in the hover condition. This variance led, in principle, to more volatile mouse movements indicating that participants in the click condition moved more impulsively than participants in the hover group. This might be caused by the loosened relationship between movement and commitment to a response: Since participants had to click the response box in order to finalize their response, an impulsive movement into a response box had no immediate consequences. As a result, participants could enter a response box while the cognitive evaluation of a trial was still not completed. Two findings support this interpretation: First, we found that participants in the click condition spent significant parts of a trial with the mouse cursor being inside a response box, which indicates that they were not fully committed to the response upon entering the response box. Second, we found a substantially higher number of dCOMs in the click group than in the hover group. A dCOM is a trajectory shape that is conflicting with core assumptions of the mouse-tracking procedure (Wulff et al., 2019): Mouse tracking is assumed to record cognitive processing through its continuous manifestation into movement (O’Hora et al., 2013; Spivey & Dale, 2006). In contrast to this assumption, dCOM trials indicate discontinuity in this manifestation, with movement being ahead of processing. More specifically, the initial movement toward the vicinity of the unchosen option in a dCOM trial is not indicative of a continuous progress of the decisional processes. Instead, it represents an overemphasis of an initial tendency, after which the decision process leading to the ultimate choice kicks in, through which the movement direction is abruptly corrected; the velocity profile of dCOM trials also suggests this pattern. The prematurity of these excessive initial movements in dCOM trials can also be inferred from our observation that movement initiation often occurred even before stimulus presentation (see also Vogel et al., 2018) and that dCOM trials had faster movement initiations than non-dCOM trials. The violation of the assumption of continuous manifestation bears practical consequences as dCOM trials bias analyses of action dynamics: When we removed dCOM trials from our analysis, we found a strong reduction of the Simon effect in horizontal mouse movements for the click group, but not for the hover group.

#### Response strategies

In the hover group, we found weaker effects in action dynamics than in the click group. Although this could be explained through the high frequency of dCOMs in the click condition, delays in movement initiation contributed to them as well: Participants were free to initiate their movement in relation to stimulus presentation and this self-paced initiation of movement led to significant delays (see Scherbaum & Kieslich, 2018). Such delays were associated with shorter movement times and more direct mouse movements. In a Simon task with pointing movement recording, Buetti and Kerzel (2009) reported equivalent associations between initiation time and movement time as well as the initial angle of movement. These findings imply that cognitive processing had already begun before movement initiation thus reducing the amount of observable competition between response alternatives.

n case of the hover condition, consequences of movement initiation times were more pronounced: The hover condition showed longer and more frequent delays in movement initiation time. This was also reflected in the groups’ ballistic velocity profile with low cursor velocity at the beginning of trials and a sudden acceleration to high cursor velocity in the final stages.

We found two distinct interindividual response strategies (see Vogel et al., 2018): Participants who initiated movement quickly exhibited curved trajectories indicating competition between responses during movement. Those who initiated movement slowly in turn exhibited mostly straight trajectory with virtually no curvature. This indicates that these participants were committed to a response before their movement began. Hence, the cognitive processing leading to this commitment could not have manifested itself into mouse movements, rendering resulting mouse trajectories unsuitable for inference of cognitive processing. We found this pattern of different response strategies especially pronounced in the hover group.

Finally, we found that premature movement initiations were more common in the click group. Participants were able to move the cursor toward the top of the screen before stimulus onset, thereby reducing the distance toward the response boxes as well as the amount of observable competition between response alternatives.

All together, we have found problems that hamper the manifestation of cognitive processing in mouse movements. Ideally, mouse-tracking data should encompass cognitive evaluation entirely—participants should only move while they are processing. In contrast, our findings reveal that participants in both conditions delayed movement initiation (albeit more pronouncedly in the hover condition), and that the participants in the click condition were prone to delayed processing.

### Response box position and hand/cursor movement ratio

Both secondary design factor manipulations revealed more subtle influences than those we found for response selection. In the manipulation of response boxes, these were either placed in the corner of the screen or in an indented, medial position. We found a reduction in mouse velocity at the end of trials when boxes were placed medially. This indicates that medially placed response boxes impede mouse movements through increased requirements in movement planning and precision.

However, we found no reduction in *x*-movements for the medially placed response boxes, which indicates that the overall impediment did not influence the continuous manifestation of cognitive processing in mouse movements. Moreover, we did not find evidence that medially placed response boxes affected participants in the click condition more than those in the hover condition.

For hand/cursor movement ratio, we wanted to determine whether participants would be able to adapt to a low hand/cursor movement ratio. We found mixed results: On the one hand, the low ratio led only to higher mouse cursor velocity at the end of a trial, indicating that participants utilized the low ratio to move faster when it was favorable to do so (i.e., when they were fully committed to a response and just had to finalize the movement as fast as possible). Additionally, this indicates that participants slowed their mouse movements down to achieve a similar velocity as with the high ratio for the most part of a trial. On the other hand, the low ratio also led to more frequent dCOMs, especially in the click condition. With the low ratio, small tendencies in mouse movements will get enlarged in cursor movements. Thus, initial movement tendencies can get over-pronounced, which is problematic when it is hard to recover from movement tendencies, as it is the case for dCOMs.

Taken together, this indicates that participants adapted to different hand/cursor movement ratio levels to some degree while they also exhibited drawbacks from amplified response tendencies. This overall pattern is in line with initial speculation in previous discussion of this design factor (M. H. Fischer & Hartmann, 2014).

### Implications

This article contributes to a developing discussion about the impact of boundary conditions of the mouse-tracking procedure on consistency and validity of resulting mouse-trajectory data (Faulkenberry & Rey, 2014; M. H. Fischer & Hartmann, 2014; Hehman, Stolier, & Freeman, 2015; Kieslich, Schoemann, Grage, Hepp, & Scherbaum, 2019; Scherbaum & Kieslich, 2018; Schoemann, Lüken, Grage, Kieslich, & Scherbaum, 2019; for hand motion tracking in general, see Song & Nakayama, 2009).

Though mouse tracking is one of the simplest methods of hand motion tracking, with fewer degrees of freedom than most other movement-tracking methods (e.g., movements are constraint to two dimensions), it still contains many potential design choices. The high impact of these design choices on the conceptual validity of mouse data—the manifestation of cognitive processes in the movement—indicates that similar design choices could have an even greater impact on data quality in other, less restricted methods of hand motion tracking.

#### Methodological implications from the perspective of affordances

When one asks for the mechanisms that lead from design choices to changes in movements, the perspective of affordances opens up an interesting space for speculation and the creation of hypotheses. From this perspective, all parameters of experimental tasks create affordances for the participant. They determine the set of possible actions and—by extension—enable/prevent specific strategies or modes of processing (see Cisek, 2007). An illustrative example is provided by research in multitasking in which the costs of processing several tasks simultaneously is investigated (Pashler, 1994). For a long time, the field focused on the question whether participants perform several tasks in parallel or in a sequential manner. It turned out that participants are actually able to execute multiple tasks in both manners (see R. Fischer & Plessow, 2015, for a review). The type of processing depends on parameters of the task (Miller, Ulrich, & Rolke, 2009) and instruction though it seems that under free circumstances participants tend to use parallel processing preferentially (Lehle & Hübner, 2009). Applying these findings to mouse-tracking studies, one can speculate that design factors create different affordances for action selection and planning, thus enabling, facilitating or discouraging different modes/strategies of task execution (see Vogel et al., 2018). This holds true for the starting procedure, the response selection method, and response box positions.

First, a static starting procedure (i.e., stimulus presentation is triggered automatically), for instance, might enable participants to evaluate the task cognitively before planning and performing the required movement to respond to it—a strategy that is not available in a dynamic starting condition (i.e., stimulus presentation is triggered by movement initiation; Scherbaum & Kieslich, 2018). Hence, the static starting procedure allows participants to choose between different strategies, which is exactly what the present study shows: Some participants tend toward the deliberate “thinking-before-moving” strategy, while some tend toward the “thinking-while-moving” strategy. Regarding the validity of mouse movements as a measure of cognitive processes, only the latter strategy allows for a continuous manifestation of cognitive processing into movement.

Second, a click response selection allows for decoupling movement from commitment to a response, whereas a hover response means that moving in a direction implies a commitment (by increasing the danger of eliciting the response). The former allows for participants to express a tentative commitment more strongly at the potential cost of increased number of changes of mind and hence dCOM trajectories. In contrast, the latter variant better fits the assumptions that underlie continuous mouse tracking: Moving the mouse toward a response location while evaluating both options is ought to be mirrored by the underlying cognitive processing, which is often illustrated as navigating through an attractor landscape with two basins of attraction. During the ongoing cognitive evaluation, evidence for both competing responses is accumulated that “pulls” the cognitive state toward the currently stronger basin of attraction. The closer the cognitive state is to an attractor, the stronger is the pull toward it (O’Hora et al., 2013; Spivey & Dale, 2006; Zgonnikov, Aleni, Piiroinen, O’Hora, & Di Bernardo, 2017). Thus, the closer the mouse is to a choice option, the more difficult it should be to turn away from it (see also Thura & Cisek, 2014). Our data indicated that this was not the case in the click group, as demonstrated by their frequent dCOM trajectories. The additional click could function as a safety net in the case of an erroneous movement to a response location, enabling action plans with a more impulsive style of movement in which the mouse cursor position does not resemble the position of the cognitive state in the attractor landscape as closely as it could be the case in the hover response selection. However, our data also indicate that the implications of the response selection are not independent of the starting condition. Since the hover condition does not offer the safety net of the click condition, participants might exploit the static start condition and the potential to delay their movement as another safety net. In fact, participants in the hover group were more likely to choose the aforementioned “thinking-before-moving” strategy (which is afforded by the static starting procedure) than were the participants in the click group. Hence, the combination of a hover response and a static starting condition also bears the potential to create a mismatch between the cognitive state and cursor position.

Third, locating the response boxes in the corners of the screen could afford more flexible movement planning, due to lower requirements in precise aiming than with the medial placement of response locations. It has been shown that movement plans for locations are prepared before movement initiation and that these plans compete with each other continuously during the choice process (see Gallivan, Chapman, Wolpert, & Flanagan, 2018, for a review). Locations themselves (e.g., the spatial separation of two response locations) have an impact on which movement plans are considered and hence express conflict while moving (Ghez et al., 1997). The more complex the available movement plans to response locations are, the more they might be incorporated into movement trajectories. If, for instance, the response locations were to be very small, this would afford a tentative approach toward it, steadily decreasing velocity the closer the mouse cursor gets to the location. In such a movement trajectory, the manifestation of cognitive processing should be heavily confounded with the demanding movement preparation and execution. This might be one factor that influenced mouse velocities toward the end of movement trajectories in our study.

From this discussion, we can draw two conclusions: First, one has to look at the affordances that are created by design factors in order to hypothesize how they influence participants’ movement strategies and hence the potential that cognitive processes manifest in the movement. Second, design factors determine the possible set of strategies for task execution. The ideal choice limits participants to exactly the one strategy that maximizes the continuous manifestation of cognitive processing in mouse movements. These two points together lead to the question: What is the ideal choice, the so-called best practice? Because work toward the best practices of mouse tracking has just begun, it is too early to stipulate definitive recommendations, especially since only a small subset of possible design factors has been investigated in previous studies and the study presented here. Nevertheless, three suggestions could be inferred on the basis of the present study:

First, regarding the method of response selection, the most impactful design factor analyzed, we make two recommendations. For designs using a static starting procedure, the click condition seems to be preferable. Although we deem the additional click required in response selection to be problematic, since it leaves room for second guessing oneself, the adverse effects demonstrated in this study for the click condition (i.e., frequent occurrence of dCOMs) were less severe than those for the hover condition (i.e., long and frequent delays in movement initiation times, preventing the manifestation of cognitive processing into mouse movements). However, with the dynamic starting procedure, delays in movement initiation times are impossible (Scherbaum & Kieslich, 2018), rendering the hover response condition preferable. Additionally, this also prevents moving significant distances before stimulus onset, which we observed in our data as well.

Second, regarding the hand/cursor movement ratio, we suggest using a high hand/cursor movement ratio, particularly if the influence of initial movement tendencies should be reduced. Note, however, that it is difficult to replicate and report the hand/cursor movement ratio accurately. This ratio depends on the resolution of the computer mouse’s sensor and the mouse settings of the operating system. Moreover, appropriate ratios vary depending on the screen’s resolution. Thus, hand/cursor movement ratios should be reported as centimeter hand movement per centimeter cursor movement measured manually with the settings used for an experiment. Additionally, screen size should be reported in order to determine the distance the mouse has to be moved in order to give a response. Maybe for these reasons, hand/cursor movement ratio is the least-reported design factor in the literature (Schoemann, O’Hora, et al., 2019).

Third, and finally, regarding the response box position, we suggest placing response boxes directly in the top corners of the screen. Although we did not find differences in action dynamics between corner and medial response boxes, the latter still proved to impede mouse movements through their higher demands, in terms of movement planning and precision. Available movement plans in mouse-tracking tasks should be kept as simple as possible.

Given the variety of design choices found in mouse-tracking studies and their potential effects on the manifestation of cognition into action, the discussion should ultimately lead to defining a standard practice of the mouse-tracking procedure. In the meantime, design choices should be reported in detail for the sake of comparability between and replication of studies. Furthermore, descriptive measures of continuous mouse data (i.e., movement densities and movement initiation times) should be analyzed and reported in order to spot possible problems regarding the quality of the mapping between cognitive processing and mouse movements. This holds especially for the frequency of dCOM trials. Awareness of the detrimental effect of discrete movement patterns (such as dCOMs) on aggregated trajectory data was present from the beginning of the rise of mouse tracking (e.g., Spivey et al., 2005). However, prevention was usually limited to distributional analyses of curvature indices (see Freeman & Dale, 2013, for an overview). Only recently has the focus shifted to the detection of discrete movement patterns—for example, the classification of individual trajectories into different types (including dCOMs) based on their shape (Kieslich et al., 2019; Wulff et al., 2019). Hence, the investigation of aggregated mouse trajectories today should at least include reporting data on dCOM trials. An exclusion of dCOM trials might also be advisable (if appropriate) since a small number of dCOM trajectories suffices to lead to disproportionately big changes in average mouse trajectories.

#### Theoretical implications for models of cognitive processing

Our study focused on how the manifestation of cognitive processing in mouse movements is influenced by the details of the procedure, which seems to imply that its implications are only methodological. However, the pattern of influences we found bears strong theoretical reverberations, considering that the shape of mouse movements has already been used to decide between different theories of cognition and decision making, namely holistic and dual-system models. In holistic (and often dynamic) models of cognitive processing, a choice is assumed to be selected through parallel evaluation of competing response alternatives over time (Spivey, 2007; see also Stillman et al., 2018). Dual-system models, in contrast, assume two distinct systems with different temporal signatures, one fast and one slow (Dhar & Gorlin, 2013; Kahneman, 2011; but see also Keren & Schul, 2009). Holistic models predict continuous mouse trajectories (a continuous competition between different responses) whereas dual-system models predict straight trajectories (initiated by the fast system) and trajectories with abrupt (i.e., discrete) course changes (initiated by the slow system). Mouse-tracking studies have provided support for both types of models, although there has been more support for holistic models overall (Stillman et al., 2018).

When aiming to decide between the two theoretical accounts, the occurrence of bimodal distributions of trajectory deviations (Freeman & Dale, 2013) or dCOMs could be seen to support dual-system accounts of cognitive processing, since they cannot be explained by holistic models. However, the present study indicates that mouse tracking not only captures cognitive processing but also its interaction with the boundary conditions of the implemented procedure. Hence, bimodal distributions and dCOMs could be caused by the way the design choices of a mouse-tracking task impact the manifestation of cognitive processing into mouse movements (see also Kieslich et al., 2019). In our data for example, the high number of dCOMs found in the click condition could in principle be regarded as a signature of dual systems being at work. However, in light of the differences in such signatures depending on the design factors, we argue that another interpretation is equally valid: The implementation of the procedure simply offered participants the response strategy of delayed processing of the presented stimulus. Hence, in order to use mouse tracking to test theoretical accounts based on trajectory shape, one should—in the sense of a strong test of a theory (Popper, 1935/2008)—consider implementing a procedure that does not in itself produce the expected shape. A test of dual-system accounts would need to utilize a set of design factors that would allow mouse movements to appear as continuous as possible. Only evidence for staged processing found in such an experiment would provide strong support for dual-system accounts of cognitive processing.

### Limitations

In the present study we investigated three design factors and their interaction with regard to their impacts on the manifestation of cognitive processing into mouse movements. Although this provided an already complex pattern of influences on the continuous manifestation of cognition into mouse movements, a single study is naturally limited in the scope of potential manipulations. Many other design factors vary between studies in the field (e.g., mouse movement instructions) and further interactions need to be investigated (e.g., click response selection with a dynamic starting procedure). Moreover, we varied each factor only with two levels. To determine acceptable levels of these factors, a gradual manipulation would be beneficial, especially in case of the hand/cursor movement ratio.

In the setup of our experiment (and in general in mouse-tracking studies that use a static starting procedure and delayed stimulus presentation), it was possible to initiate movements before stimulus presentation. This could be seen as a methodological flaw: Though such early movements cannot be ascribed to cognitive processing (pertinent to the task), they are usually compared with movements that were initiated at or after stimulus presentation nonetheless. We deliberately decided to implement this methodological problem in our setup in order to emulate the setups of several mouse-tracking studies for which we wanted to study the design factors of interest. In other studies in our lab, we have prevented such premature movement initiation by implementing a dynamic starting procedure in which movement initiation and stimulus onset are linked. Studying this as an extra design factor revealed a higher overall quality in movement (Scherbaum & Kieslich, 2018). Hence, this dynamic start procedure should be implemented by more studies if their general setup allows for it. Alternatively, these premature movements could be prevented by removing the click–stimulus interval, or signaling premature movement initiation as an error to the participant.

A final concern relates to the generalization of our findings to other processes studied with mouse tracking: The Simon effect has shown a robust manifestation of cognitive processing in mouse movements (Scherbaum et al., 2010; Scherbaum & Kieslich, 2018). However, it is unclear how stable the manifestation is for other, presumably more complex processes—for example, value-based decision making (Koop & Johnson, 2011; Scherbaum et al., 2016; Sullivan et al., 2015). First evidence from intertemporal choice tasks (Schoemann, Lüken, et al., 2019) has indicated that design factors might have a stronger influence for more complex processes. Currently, one can only speculate that even more complex processes—for example, decisions in moral dilemmas (Koop, 2013), might show similarly strong, or even stronger, influences of design factors.

### Conclusion

With methods of hand motion tracking, researchers aim to gain insights of cognitive processing by utilizing the continuous manifestation of cognition into movement. For this reason, such methods as mouse tracking have been regarded as powerful tools for studying changes in cognitive states over time. In this study, we added to the growing body of evidence that the validity of the inference from movement to cognition is dependent on the design factors of the mouse-tracking procedure. We investigated different levels of three design factors that could impede the manifestation of cognition into movement, as reflected in discrete effects, continuous measures, and movement consistencies. Hence, it is crucial to make deliberate and, if possible, informed design choices in mouse-tracking studies. By working toward a standard setup of the mouse-tracking procedure, researchers would be enabled to utilize the manifestation of cognition in action to its full potential.

## Electronic supplementary material

ESM 1 (PDF 655 kb)

## Footnotes

1 Conversely, movement influences cognition, as well. For instance, the higher the distance already traveled toward an option (which can serve as an indicator of the degree of commitment to it), the lower the chance of a redirection to a competing response (Thura & Cisek, 2014).

2 The shortest distance between the start box and the response boxes (placed in the corners of the screen) was 34.1 cm. Thus, the required minimal hand movements to reach the response box were 3.1 and 5.4 cm for the low and the high hand/cursor movement ratio, respectively.

3 For an alternative analysis approach that aims to identify changes of mind based on trajectory shapes, please see Wulff et al. (2019).

4 Note that in more recent studies—for example, Frisch, Dshemuchadse, Görner, Goschke, and Scherbaum (2015)—another method of the dynamic starting procedure was employed that also considered the direction of movement: Participants had to move 50 pixels upward to trigger stimulus onset.

5 Similarly, negative correlations were found between initiation time and the average deviation of mouse movements (see the supplementary material), confirming the assumption that long initiation times were associated with direct movements.

6 We did not analyze *x*-movements for the design factor hand/cursor movement ratio, since the low ratio resulted in significantly shorter response times than did the high ratio. Such differences in response times would lead to a distorted stretching of the faster trials in time normalization of the mouse data. Differences in the amplitudes of mouse trajectories are unaffected by this distortion, which is why we still analyzed mouse cursor velocities (see the supplementary material for a response time analysis and plot of *x*-movements).
