# Supplementary material for: Lost to translation: How design factors of the mouse-tracking procedure impact the inference from action to cognition
Source: Atten Percept Psychophys. 2019 Nov 5;81(7):2538–57. doi: 10.3758/s13414-019-01889-z (PMC6848042; doi:10.3758/s13414-019-01889-z)
Supplement: Supplementary file 1 — (PDF 655 kb) [file 13414_2019_1889_MOESM1_ESM.pdf]

Supplementary Material

Lost to translation: How design factors of the mouse-tracking procedure impact the inference from action to cognition

Tobias Grage[†], Martin Schoemann[†], Pascal J. Kieslich[‡], & Stefan Scherbaum[†]

† Department of Psychology, Technische Universität Dresden, Dresden, Germany

‡ Department of Psychology & Mannheimer Zentrum für Europäische Sozialforschung (MZES), School of Social Sciences, University of Mannheim, Mannheim, Germany

---

1 Discrete effects

1.1 Response times

Here, we define response times (RT) as the time between stimulus onset and reaching a response box. The additional time in the click response selection between reaching the response box and clicking into it was ignored for our analyses.

We performed a four-way mixed analysis of variance (ANOVA) on RT with the between-subject factor Response Selection (hover vs. click) and the within-subject factors Congruency (congruent vs. incongruent), Hand/ Cursor Movement Ratio (low vs. high) and Response Box Position (corner vs. medial)[1]. The ANOVA revealed a significant main effect for Congruency, $F(1, 36) = 127.91, p < .001, \eta_p^2 = .78$. Response times were higher for incongruent trials (M = 624.9 ms, SD = 63.1 ms) than for congruent trials (M = 583.2 ms, SD = 70.3 ms), replicating the Simon effect. There was a significant main effect of Hand/ Cursor Movement Ratio, $F(1, 36) = 95.46, p < .001, \eta_p^2 = .73$: When Hand/ Cursor Movement Ratio was low, response times were reduced (low: M = 573.5 ms, SD = 63.1 ms; high: M = 634 ms, SD = 73.8 ms). Additionally, there was a significant interaction between Congruency and Response Box Position, $F(1, 36) = 11.55, p = .002, \eta_p^2 = .24$. Paired samples t-tests for each level of Congruency revealed

[1]We want to point out that in preparation of analyses of variance (ANOVA) across different measures, we found heterogeneous variances in different factor levels between the hover and the click condition as determined by Levene's Test. Since this violation of the ANOVA's assumption of equal variances is associated with an inflation of the Type I error (Harwell, Rubinstein, Hayes, & Olds, 1992) a conceptually equivalent linear mixed model analysis was calculated for each ANOVA affected. In every case, both the linear mixed model and the ANOVA yielded the same significant effects. Hence, we will still report the results of the ANOVA in the following. The measures affected by this were RTs, maximum deviation, and discrete changes of mind.

that incongruent trials differ across Response Box Positions, $t(37) = 2.22$, $p = .033$, $d_z = 0.36$: Incongruent trials were slower with a medial Response Box Position ($M = 629.7$ ms, $SD = 62.9$ ms) than with a corner Response Box Position ($M = 619.9$ ms, $SD = 66.1$ ms). There was no significant difference between Response Box Positions for congruent trials, $t(37) = 0.246$, $p = .807$. Finally, there was a significant interaction between Congruency and Response Selection, $F(1, 36) = 12.61$, $p = .001$, $\eta_p^2 = 0.26$. A two samples t-test revealed a significantly larger Simon effect for the click Response Selection ($M = 54.9$ ms, $SD = 26.3$ ms) as compared to the hover Response Selection (hover: $M = 28.5$ ms, $SD = 19.1$ ms), $t(36) = 3.54$, $p = .001$, $d_z = 0.57$. Two separate t-tests were calculated to determine the effect sizes of each Simon effect with the click Response Selection showing a considerably larger effect size: click, $t(36) = 10.42$, $p = < .001$, $d_z = 1.69$; hover, $t(36) = 3.68$, $p = .002$, $d_z = 0.6$. There were no other significant effects (all ps $\geq .232$).

We found the basic Simon effect in RTs across all design factors. Furthermore, participants in the click condition exhibited stronger Simon effects as participants in the hover condition. In incongruent trials, medial response boxes led to higher response times than corner response boxes. Lastly, participants apparently made use of the low hand/ cursor movement ratio when it was available and responded faster.

1.2 Maximum movement deviation

As a discrete measure for the mouse movements, we calculated the maximum deviation of the actual movement trajectory from the direct line between start box and response box for each trial. For this, we used the individual start and endpoint of each trial[2]. We performed the same four-way mixed ANOVA for all design factors on maximum deviation as above on RT. It revealed significant main effects for every design factor (see Table 1). Additionally, all two-way interactions between the factors Congruency, Response Selection, and Hand/ Cursor Movement Ratio were significant: The interaction between Congruency and Response Selection, $F(1, 36) = 15.81$, $p < .001$, $\eta_p^2 = .31$, was followed up by a post hoc Welch's t-test on the corresponding Simon effects. The effect is significantly larger in the group with click Response Selection ($M = 146.91$ pixel, $SD = 81.34$ pixel) than with hover ($M = 63.62$ pixel, $SD = 41.78$ pixel), $t(26.88) = 3.97$, $p < .001$, $d_z = 0.64$. The interaction between Response Selection and Hand/ Cursor Movement Ratio, $F(1, 36) = 8.13$, $p = .007$, $\eta_p^2 = .18$, was followed up by separate t-tests for both Response Selection groups: Hand/ Cursor Movement Ratio only had an impact on maximum deviation in the click Response Selection group (low: $M = 252.87$ pixel, $SD = 96.53$ pixel, high: $M = 223.76$ pixel, $SD = 84.03$ pixel), $t(18) = 3.78$, $p = .001$, $d_z = 0.87$, but not in the hover group (p = .312) Lastly, there was a significant

[2] A better way of comparing deviation would be to add a control condition in which only movement characteristics are captured in order to calculate an average path to compare movement trajectories against.

Table 1: Main effects of four-way mixed ANOVA on maximum deviation. The effect of Hand/ Cursor Movement Ratio was explained by its interaction with Response Selection

Design factor	$F(1,36)$	p	η_p^2	M_1 (SD_1)	M_2 (SD_2)
Congruency	100.92	< .001	.74	congruent: 137.34 (69.59)	incongruent: 242.61 (125.15)
Response Selection	14.10	< .001	.28	hover: 139.84 (70.24)	click: 238.39 (88.95)
Hand/ Cursor Movement Ratio	14.94	< .001	.29	low: 197.34 (101.78)	high: 180.32 (86.85)
Response Box Position	7.01	< .001	.16	corner: 194.79 (97.14)	medial: 183.46 (91.70)

Note: Means and standard deviations measured in pixel.

interaction between Hand/ Cursor Movement Ratio and Congruency, $F(1,36) = 7.53, p = .009$, $\eta_p^2 = .17$. Comparing Simon effects, the low movement ratio exhibited a significantly stronger effect, $t(37) = 2.62, p = .013, d_z = 0.43$; low ratio: $M = 112.18$ pixel, $SD = 82.5$ pixel; high ratio: $M = 98.37$ pixel, $SD = 73.52$ pixel. No other effects were significant (all ps ≥ 0.521).

Similar to the analysis of response times, stable Simon effects on movement deviation were found as well as differences between design factors.

2 Movement strategies

Table 2: Correlations of movement initiation time and average deviation on subject level.

Hover			Click		
r	95% CI	p	r	95% CI	p
-.97	[-.91, -.99]	< .001	-.77	[-.48, -.91]	< .001

Note: Correlations were calculated by correlating participants' means for initiation time with participants' means for average deviation.

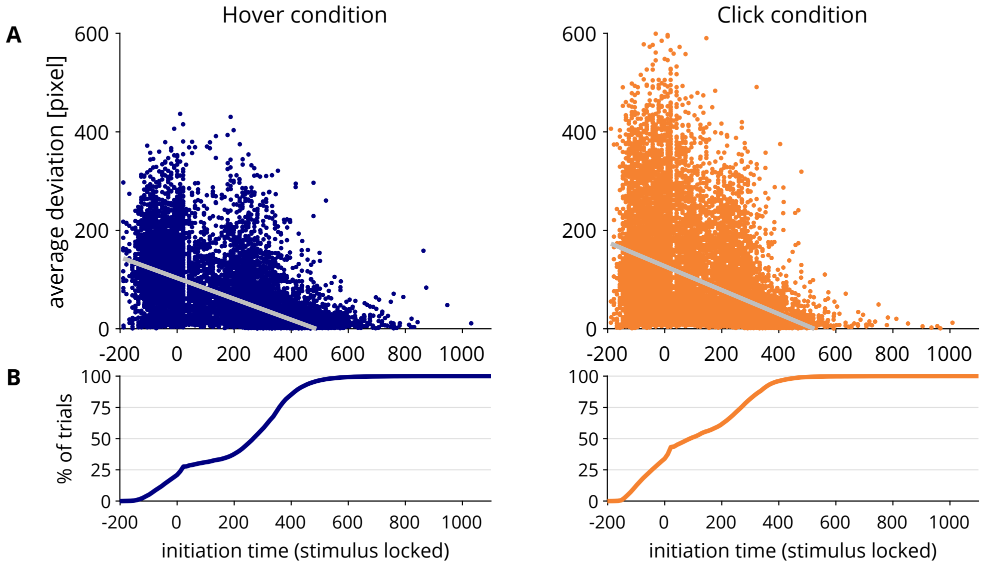

Figure 1: Scatterplots of initiation time and average deviation (A) separate for hover (left) and click (right) response selection (pooled data). Cumulative percentage of trials is shown as a function of initiation time (B).
Note: Solid grey lines in (A) depict least-squares fit.

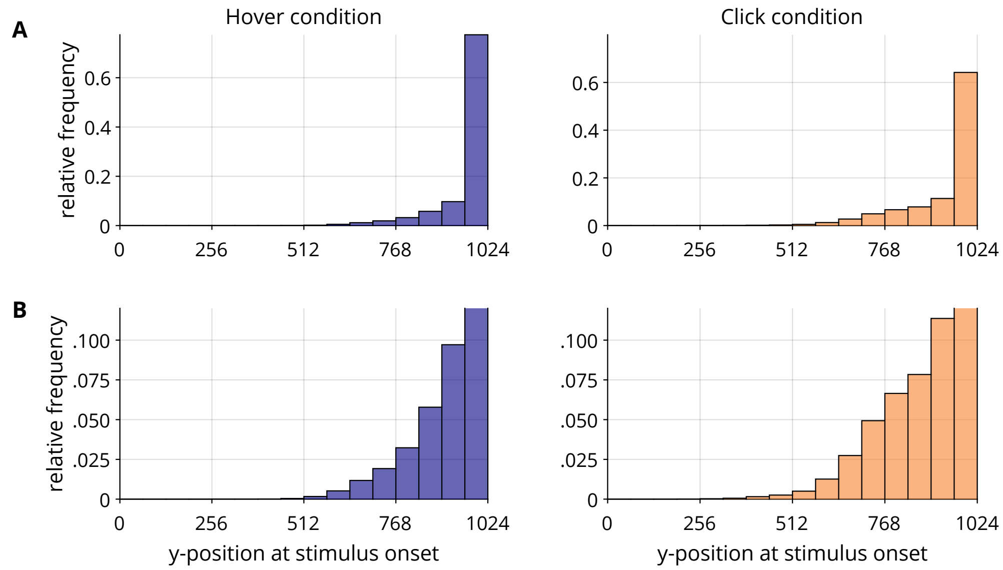

Figure 2: Vertical mouse cursor position in pixel at time of stimulus onset. Y-position ranges from top of the screen (0 pixel) to bottom of the screen (1024 pixel). (A) original view, (B) zoomed view, cutting the last bin (960–1024)

3 Response box position and hand/ cursor movement ratio

The following trajectory data was first averaged within and then across participants.

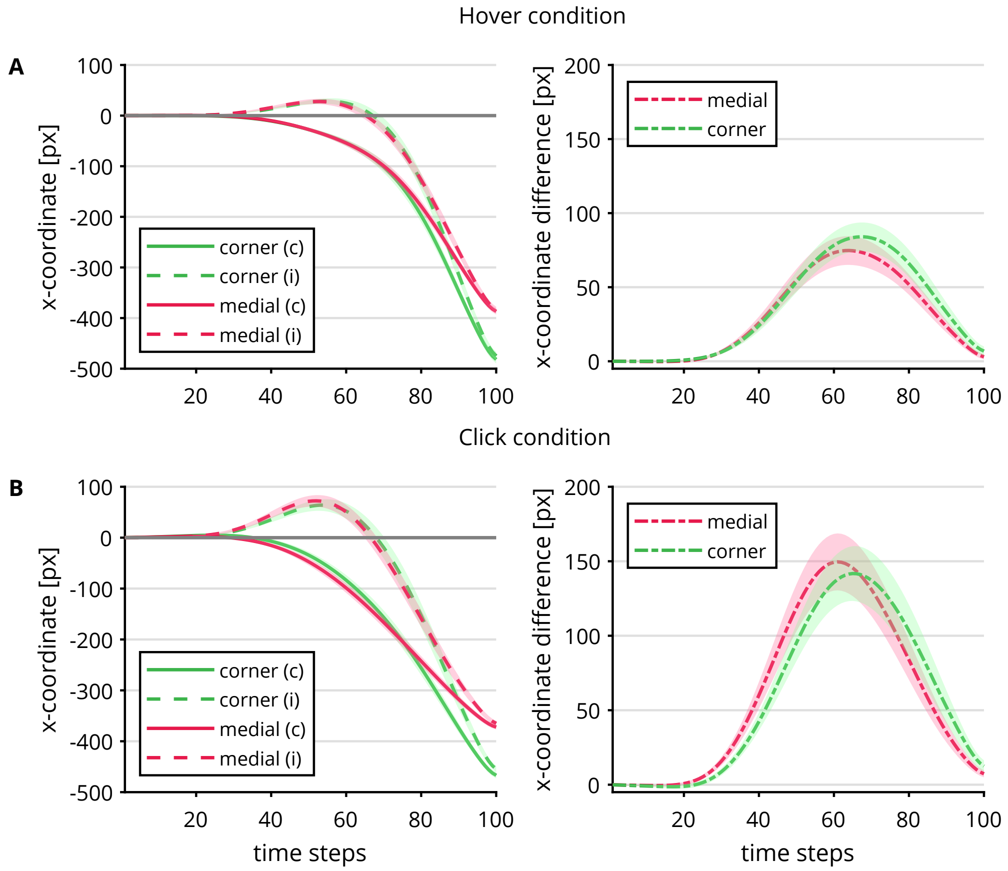

Figure 3: Mouse trajectory data along the x-axis per time normalized step of incongruent (i) and congruent (c) trials (left) and incongruent-congruent contrast (right) for levels of response box positions in the hover condition (A) and the click condition (B)
Note: Shaded confidence bands depict standard errors.

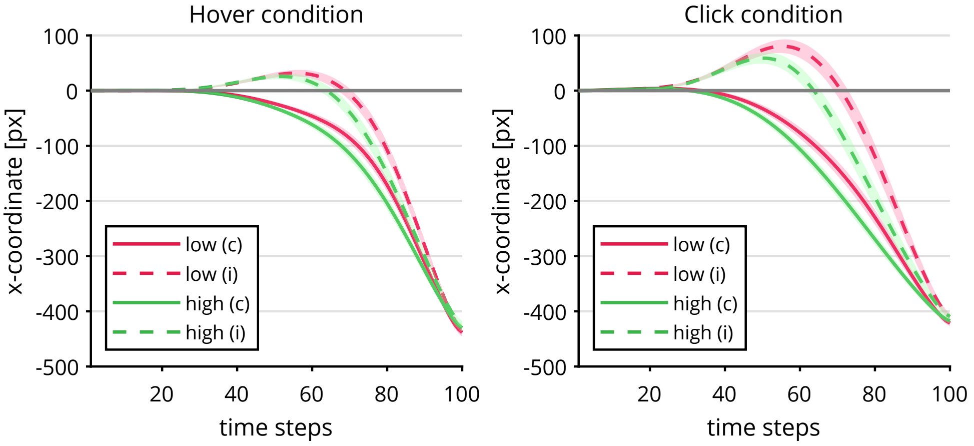

Figure 4: Mouse trajectory data along the x-axis per time normalized step of incongruent (i) and congruent (c) trials in the hover condition (left) and the click condition (right) separately for low and high hand/ cursor movement ratios.
Note: Shaded confidence bands depict standard errors.

References

Harwell, M. R., Rubinstein, E. N., Hayes, W. S., & Olds, C. C. (1992). Summarizing Monte Carlo Results in Methodological Research: The One- and Two-Factor Fixed Effects ANOVA Cases. *Journal of Educational Statistics*, *17*(4), 315. doi: 10.2307/1165127
